# Pregnant women utilization of dental services: still a challenge in low resource setting

**DOI:** 10.1186/s12903-021-01746-2

**Published:** 2021-08-05

**Authors:** Chidozie Onwuka, Chidinma Ifechi Onwuka, Emeka Ifeanyi Iloghalu, Peter Chukwudi Udealor, Euzebus Chinonye Ezugwu, Ifeanyi Emmanuel Menuba, Emmanuel Onyebuchi Ugwu, Chinyere Ututu

**Affiliations:** 1 Oral and Maxillo-Facial Surgery Department, King Khalid University, Abha, Saudi Arabia; 2 Obstetrics and Gynaecology Department, University of Nigeria Nsukka/University of Nigeria Teaching Hospital, Enugu, Nigeria; 3 Department of Child Dental Health, University of Port Harcourt Teaching Hospital, Rivers State, Nigeria

**Keywords:** Dental consultation, Pregnancy, Predictors, Enugu, Women

## Abstract

**Background:**

Poor oral health in pregnancy can be associated with poor pregnancy outcome, however, dental consultation among pregnant women appears to be low.

**Methods:**

This was a questionnaire-based study of 413 women who attended the antenatal clinic of University of Nigeria Teaching Hospital (UNTH), Ituku/Ozalla, Enugu. The information obtained was analyzed using SPSS version 22. A *p*-value of less than 0.05 was considered statistically significant.

**Results:**

Only 36 (8.7%) of the respondents had dental consultations in index pregnancy for complaints such as tooth ache and decay (66.7%) and pain as well as swelling of the gum (33.3%). The most common reason given for not visiting a dentist during the index pregnancy was the visit not being relevant to their pregnancy outcome (69.2%). After counseling them, only 249 (60.3%) agreed to have dental consultation during subsequent pregnancies. The relationship between visiting the dentist and place of residence (< 0.001), occupation (0.019) and frequency of brushing/ changing of brush (0.005, < 0.001 respectively) were statistically significant.

**Conclusion:**

The prevalence of dental consultation during pregnancy is very low. Pregnant women should be encouraged to have routine dental consultation with oral health counseling and check-up incorporated as part of routine antenatal care.

## Background

Pregnancy is known to be associated with various physiological changes in women which may include changes in the oral cavity. These changes may predispose them to periodontal diseases and other oral lesions, and could even worsen an already existing lesion [1–3]. In order to prevent the development of oral health related diseases in pregnant women and their young children; a routine dental check-up is highly recommended as part of antenatal care [1–3]. Good oral health during pregnancy is known to be beneficial to the mother and the baby, and helps to prevent cavities in young children [4, 5]. Adverse effects linked with maternal poor oral health such as periodontal diseases include preterm birth and low birth weight [4]. The maternal cariogenic organism can be transmitted from mother to child even after childbirth [2, 5]. Children of women with poor oral health in pregnancy are known to be at risk of developing dental caries which is the most common but preventable chronic disease in some developed countries like United States [1].

Pregnant women are more likely to suffer from dental caries when compared with the non-pregnant ones because of frequent consumption of sugary foods and drinks to alleviate nausea and vomiting; also because of frequent vomiting in first trimester [6]. However, most pregnant women do not seek dental consultation or treatment due to fear of potential harm to themselves or their babies [4]. Oral health screening is not routinely done in most antenatal clinics in Nigeria and in many developing countries of the world. Usually pregnant women are referred to the dentists only when they complain of dental related problems [4]. It is advocated that women’s health care providers should educate pregnant women, promote good oral health care and reassure women of the importance and safety of oral health care in pregnancy [1, 5]. These interventions in oral health services may help improve the oral health of the mother and her baby [1]. Pregnancy is also a time where education and preventive programs can be established since pregnant women are more receptive to information about themselves and their babies’ health thereby adopting good healthcare practices [3]. Due to the importance of oral health care in pregnancy, the American College of Obstetricians and Gynaecologists (ACOG), the American Dental Association and some other organizations in 2012 issued a consensus statement as a guideline for the Obstetricians and dentists [1]. This guideline includes taking good oral health history, examination of the mouth for oral problems, advise pregnant women on oral health, the need not to postpone or avoid oral health care in pregnancy, schedule consultation with the dentist as early as possible and encourage good oral health behaviors during pregnancy amongst other guidelines [1].

Although dental care is recommended during pregnancy, it has been shown that there is low demand for dental services worldwide ranging from 27 to 53% with majority presenting because of dental pain [3]. A study done in Nigeria reported that only 27.9% of pregnant women visited the dentist for dental care during pregnancy [7] while a Tanzanian study reported similar prevalence of 31.8% [4].

Despite the importance of oral health in pregnancy, there is limited data on dental consultation and factors that might affect dental services utilization among pregnant women in Enugu, south-eastern Nigeria. Assessing the rate of utilization of dental service by pregnant women and factors that may constitute a barrier to its utilization, hopefully will help in policy decision making aimed at improving the uptake and utilization of dental services by pregnant women in the region and in Nigeria at large. The study is aimed to determine the prevalence of routine dental consultation among pregnant women and to identify factors that affect the utilization of dental services in pregnancy.

## Methods

This was a cross-sectional study of pregnant women attending antenatal clinic at University of Nigeria Teaching Hospital (UNTH), Ituku/Ozalla, Enugu from January 2018 to May 2018. UNTH is one of the tertiary hospitals in the south-east. The Obstetrics and Gynaecology department of the hospital offers antenatal, intra-partum, postnatal and gynaecological services to women in Enugu state and the neighboring states such as Anambra, Abia, Imo, Ebonyi and Benue State. Antenatal care is offered to pregnant women at the antenatal clinic on a daily basis from Monday to Friday. Further details of the antenatal clinic and attendance are as in previous studies [8–10]. All consenting pregnant women attending the antenatal clinic within the study period were consecutively recruited for the study using a convenient sampling method. However, pregnant women who did not give consent were excluded from the study. Using an interviewer administered, semi structured questionnaire adapted from previous studies [11, 12], relevant data were obtained from the participants after obtaining their written informed consent. These data included: the women’s socio-demographic characteristics, history of dental consultation in index pregnancy, the number of times that the respondent visited the dentist, the reason for the dental consultation and the person that initiated the visit to the dentist. Other information sought included: reasons for not having dental consultation in index pregnancy and the willingness to have a dental consultation in subsequent pregnancy even after counselling. Dental consultation was defined as having visited the dentist in index pregnancy for any reason.

The sample size was calculated using the formula n = Z^2^pq/d^2^ where *p* was set as 50% [13]. The calculated minimum sample size was 384, however with 10% attrition rate, 422 participants were recruited for the study.

Data was analyzed using the Statistical Package for the Social Sciences (SPSS) software, version 22.0, IBM SPSS, Chicago, Illinois. Analysis was both descriptive and inferential. Logistic regression and chi square were used to analyze discreet variables. A *p*-value of less than 0.05 was considered statistically significant.

The ethical approval for the study was obtained from UNTH institutional Ethics review board and the research was carried out in accordance with the guidelines and regulations of the ethics board.

## Results

Of the 422 participants recruited for the study, 97.9% (n = 413) had complete data and formed the basis for analysis. The remaining 2.1% (n = 9) of participants were excluded due to incomplete data.

The mean age of the participants was 29.71 years. Majority of them were married (97%), multiparous (56.2%), Christians (96.7%), with at least secondary level of education (98.1%) and lived in the urban areas of Enugu (84.5%). About two-fifths of the participants (40%) were salary earners. Further details on the socio-demographic characteristics of respondents are provided in Table 1.

**Table 1:** Socio-demographic characteristics of the participants

	Frequency	Percentage (%)
**Age group**		
20–24	69	16.7
25–29	139	33.7
30–34	142	34.4
35–39	47	11.4
≥ 40	16	3.9
**Residence**		
Urban	349	84.5
Rural	64	15.5
**Occupation**		
Trader	83	20.1
Unemployed	75	18.2
Student	86	20.8
Salary earner	165	40.0
Farmer	4	1.0
**Religion**		
Christianity	397	96.7
Muslim	8	1.9
Traditional	4	1.0
Others	4	1.0
**Educational status**		
Primary	8	1.9
Secondary	81	19.6
Tertiary	324	78.5
**Parity**		
0	157	38.0
1–4	232	56.2
> 4	24	5.8

The prevalence of dental consultation in this study was 8.7% (N = 36/413). None of the participants had routine preventive dental consultation in index pregnancy. All the dental consultation recorded by the respondents were due to dental health issues in index pregnancy.

About 23.0% (n = 95/413) of the participants had dental health issues in index pregnancy. Of these, only about one-third of them (37.9%, n = 36/95) visited the dentist for dental care, while majority did not. The majority of those that accessed dental care were referred to the dentists by the obstetricians (83.3%, n = 30/36), four of them (11.1%) were referred by nurses and while the remaining two (5.6%) were self-referrals. The most common reason for visiting the dentist was tooth ache (Table 2).

**Table 2:** Reasons for dental consultation in index pregnancy

	Frequency (n=36)	Percent (%)
Tooth decay	8	22.2
Toothache	16	44.5
Pain/gum swelling	12	33.3
Excessive salivation	0	0.0
Routine dental consultation	0	0.0

Twenty two (61.1%) of them visited the dentist only once while 14 (38.9) visited twice or more.

Notably, majority of the participants that had dental problem did not seek dental care (62.1%, n = 59/95). The reasons given for not seeking dental care in index pregnancy were; feeling that dental problem is not relevant to their pregnancy outcome (69.2%), fear of harming the baby (11.7%), cost of care (9.5%), busy schedule (8.5%), and fear of the dentist (1.1%).

After counseling, almost two-third of the participants (60.3%, n = 249) were willing to have dental consultation during their subsequent pregnancies.

The relationship between visiting the dentist and age (*p* = 0.056) and educational status (*p* = 0.086) were not statistically significant. Whereas there was a significant relationship between visiting dentist and the participants place of residence (*p* =  < 0.001), occupation (*p* = 0.019) and religion (*p* =  < 0.001). Table 3.

**Table 3:** Association between socio-demographic characteristics and dental consultation in index pregnancy

	Visited the dentist			
	Yes n (%)	No n (%)	*p* value	RR (95% CI)
**Age group**				
20–24	8 (11.6)	61 (88.4)	0.056	
25–29	16 (11.5)	123 (88.5)		
30–34	12 (8.5)	130 (91.5)		
35–39	0 (0)	47 (100.0)		
≥ 40	0 (0)	16 (100.0)		
**Residence**				
Urban	20 (5.7)	329 (94.3)	< 0.001	0.2(0.09–0.38)
Rural	16 (25.0)	48 (75.0)		
**Occupation**				
Trader	4 (4.8)	79 (95.2)	0.019	
Unemployed	4 (5.3)	71 (94.7)		
Student	16 (18.6)	70 (81.4)		
Salary earner	12 (7.3)	153 (92.7)		
Farmer	0 (0.0)	4 (100)		
**Religion**				
Christianity	32 (8.1)	365 (91.9)	< 0.001	
Muslim	0 (0.0)	8 (100.0)		
Traditional	4 (100.0)	0 (0.0)		
Others	0 (0.0)	4 (100.0)		
**Educational status**				
Primary	(0.0)	8 (100.0)	0.086	
Secondary	12 (14.8)	69 (85.2)		
Tertiary	24 (7.4)	300 (92.6)		

Participants that reported brushing once a day were less likely to visit the dentist than those that brushed twice or more (RR = 0.3, CI 0.15–0.73, *p* value = 0.01). Also participants that changed their brush at more than three month interval were three times more likely to visit the dentist for a dental problem (RR = 3.33, CI 1.71–6.47, *p* value < 0.01). This is shown in Table 4.

**Table 4:** Association between oral health practices and dental consultation in index pregnancy

	Visited the dentist			
	Yes n (%)	No n (%)	*p* value	RR (95% CI)
**Frequency of brushing/day**				
Once a day	8 (4.3)	176 (95.7)	0.01	0.3 (0.15–0.73)
Twice or more a day	28 (12.2)	201 (87.8)		
**Frequency of change o﻿f brush**				
> 3 monthly	24 (15.5)	131 (84.5)	< 0.01	3.33 (1.71–6.47)
3 monthly	12 (4.7)	246 (95.3)		

## Discussion

The prevalence of dental consultation in this study was 8.7%. Although it is recommended that women should have dental care while pregnant, studies have shown that dental services utilization among pregnant women has remained very poor [14–16]. This trend appears to be a global one. A study done in the United States showed that less than half of pregnant women reported visiting a dentist [15]. Similar studies have also shown low dental consultation among pregnant women ranging from 13.7% to 35% [7, 11, 12, 16–19]. The prevalence reported in this current study appears to be the lowest and may be a reflection of the low level of awareness of the importance of good dental hygiene in promoting better obstetric outcome, as well as low resources available to the participants.

The most common dental complaint that necessitated dental consultation in our study was toothache. This was at variance to an earlier report by Bashiru and Ilochonwu in Port Harcourt, Nigeria where excessive salivation was the most common oral condition [7] while an Australian study found that gingival bleeding was the most common [18]. Excessive salivation was not reported at all in our study. The reason for the difference may be that our women considered excessive salivation as normal during pregnancy [7].

Although about one fifth of the participants had dental problems, majority of them did not consult the dentists. The most common reason for not visiting the dentists in the current study was the participants thought it was not relevant to their pregnancy outcome and therefore felt there was no need for it. This was one of the reasons noted in a previous study [11]. Oral problems (such as tooth decay, gum bleeding) are believed by most women to be part of the pregnancy changes and this belief serve as barrier to dental consultation [3, 7, 19].

Another reason for non-utilization of dental services was the fear of harm to the unborn child. A similar study showed that majority of the women believed that receiving dental treatment while pregnant could have negative effects on their pregnancy outcome [16–18].

Cost of dental treatment was another reason for the low dental consultation in our study. This was in consonant with previous study [3]. That is why health insurance is very important since it has been associated with better use of dental services [14, 18].

Other reasons for not visiting the dentist during pregnancy included; busy schedule and fear of the dentists. These barriers to the utilization of dental services while pregnant have been reported in a similar study [3].

Brushing twice daily was significantly associated with dental consultation. Similar study had shown that there is higher dental consultation among women who brushed regularly [11]. This may be attributed to the fact that women who brushed regularly may likely have better information and understand the need for dental consultation. Women who reported good oral care were more likely to have regular dental check-up [19]. Other factors associated with dental consultation include: place of residence, occupation, religion and frequency of change of toothbrush (*p* < 0.005).

As shown in the current study, less than half of the women that required dental consultation actually visited the dentist. Dental services may actually be under-utilized probably due to unawareness on the part of the pregnant women as well other barriers earlier listed. It has also been reported that low dental consultation may be attributed to some dentists and obstetricians because they are not counseling their patients about the importance of dental care during pregnancy [20]. This may be because they are not aware of antenatal oral health guidelines thereby not recommending dental care during pregnancy [20]. A previous study showed that antenatal care giver hardly advised pregnant women on good oral hygiene maintenance and routine dental consultations nor checked the oral status of these women [21]. Management of routine and dental emergencies can be denied by dentists because of misconceptions which can be corrected by continuous medical education [2]. Nurses also have a role to play in oral health care of pregnant women. A previous study showed that though the nurses had limited knowledge about oral health care; they had good attitudes [6]. There may be need for more collaboration between the antenatal care providers, the dentists and other health workers to ensure that pregnant women need not postpone or avoid oral health care in pregnancy, consult the dentist as early as possible and encourage good oral health behaviors during pregnancy.

None of the participants in our study had routine dental consultation. They only visited the dentist because they had dental complaints. Majority of them where referred by their Obstetricians after complaining of dental problem. Counselling is also essential as seen in our study where more than half agreed to have dental consultation in their subsequent pregnancies. This counselling should be done more by the obstetricians during antenatal period. This shows that obstetricians have a lot to do in order to improve the oral health of pregnant women. Further study on the knowledge and practices of obstetricians concerning oral health among pregnant women would be beneficial.

This study has some limitations. Convenient sampling method was used to select the participants, and this may affect the generalizability of the study result to the entire population. Also the study was based on self-reported information and may be prone to bias. However, this was minimized by the administration of the questionnaires by trained interviewers.

## Conclusion

The prevalence of dental consultation during pregnancy is very low. Pregnant women should be encouraged to have routine dental consultation with oral health counseling and check-up incorporated as part of routine antenatal care.

## Data Availability

Data of the study will be shared upon request to the corresponding author.

## Abbreviations

UNTH: University of Nigeria Teaching Hospital
SPSS: Statistical Package for Social Sciences
IBM: International business Machines Corporation
